# A Meta-Analysis of Proton Pump Inhibitor Use and the Risk of Acute Kidney Injury: Geographical Differences and Associated Factors

**DOI:** 10.3390/jcm12072467

**Published:** 2023-03-24

**Authors:** Cheng Ta Han, Md. Mohaimenul Islam, Tahmina Nasrin Poly, Yu-Chun Lu, Ming-Chin Lin

**Affiliations:** 1 Department of Neurosurgery, Shuang Ho Hospital, Taipei Medical University, New Taipei City 23561, Taiwan; 2 Taipei Neuroscience Institute, Taipei Medical University, Taipei 11031, Taiwan; 3 International Center for Health Information Technology, College of Medical Science and Technology, Taipei Medical University, Taipei 11031, Taiwan; d610106004@tmu.edu.tw (M.M.I.);; 4 Graduate Institute of Biomedical Informatics, College of Medical Science and Technology, Taipei Medical University, Taipei 11031, Taiwan

**Keywords:** proton pump inhibitor, acute kidney injury, chronic kidney disease, meta-analysis

## Abstract

Proton pump inhibitors (PPIs) are widely prescribed in medical practice for the treatment of several gastrointestinal disorders. Previous epidemiology studies have reported the association between PPI use and the risk of AKI, although the magnitude of the association between PPIs and the risk of acute kidney injury (AKI) remains uncertain. Therefore, we conducted a meta-analysis to determine the relationship between PPI therapy and the risk of AKI. We systematically searched for relevant articles published before January 2023 on PubMed, Scopus, and Web of Science. In addition, we conducted a manual search of the bibliographies of potential articles. Two independent reviewers examined the appropriateness of all studies for inclusion. We pooled studies that compared the risk of AKI with PPI against their control using a random effect model. The search criteria based on PRISMA guidelines yielded 568 articles. Twelve observational studies included 2,492,125 individuals. The pooled adjusted RR demonstrated a significant positive association between PPI therapy and the risk of AKI (adjusted RR 1.75, 95% CI: 1.40–2.19, *p* < 0.001), and it was consistent across subgroups. A visual presentation of the funnel plot and Egger’s regression test showed no evidence of publication bias. Our meta-analysis indicated that persons using PPIs exhibited an increased risk of AKI. North American individuals had a higher risk of AKI compared to Asian and European individuals. However, the pooled effect from observational studies cannot clarify whether the observed association is a causal effect or the result of some unmeasured confounding factors. Hence, the biological mechanisms underlying this association are still unclear and require further research.

## 1. Introduction

Kidney disease is one of the most common global health challenges, affecting more than 37 million Americans [1]. Acute kidney injury (AKI) and chronic kidney disease (CKD) are two common types of kidney disease. AKI is known to be a major cause of global morbidity and mortality [2,3]. However, the prevalence of AKI is rapidly increasing, especially among people living in developing countries [4]. AKI is also associated with a substantial economic burden due to increased hospitalization costs, ranging from USD 5.4 to USD 24.0 billion [5]. Therefore, attention has focused on identifying the potential risk factors of AKI to reduce the burden. Previous evidence has reported that the risk factors associated with new-onset AKI include age, hypertension, diabetes mellitus, chronic heart diseases, CKD, and nephrotoxic drugs [6,7,8,9,10].

Proton pump inhibitor (PPI) is a widely prescribed medication in patients with gastric disorders [11,12]. While the short-term use of PPI promises certain benefits, previous evidence has recently brought public attention to its adverse events in kidney diseases [13,14,15]. However, the relationship between PPI use and the risk of AKI is poorly explored. A significant number of studies found a pathophysiological association between PPI and acute interstitial nephritis (AIN) [16,17,18] and CKD [19,20], which leads to the development of AKI. Recent epidemiological studies have shown positive associations between PPI use and the risk of AKI [21,22], although several previous studies found no association, which makes the findings inconsistent [23,24]. Therefore, an overall effect size calculation is needed in order to make the most effective clinical decisions.

To our knowledge, no comprehensive systematic review and meta-analysis of the association between the use of PPI and the risk of AKI has been published so far. Therefore, we performed an updated systematic review and meta-analysis of observational studies to determine whether the use of PPI is associated with an increased risk of AKI when compared to non-PPI users. In our study, we examined the risk of AKI among PPI users based on study design, region, quality of the study, and PPI types. This study might help clinicians to weigh the risk against overall benefits and provide clinical guidance.

## 2. Methods

This systematic review and meta-analysis were conducted based on the Preferred Reporting Items for Systematic Reviews and Meta-Analyses (PRISMA) guidelines [25,26]. A review protocol was not drafted.

### 2.1. Search Strategy and Selection Criteria

We searched electronic databases, such as PubMed, Scopus, and Web of Science, before January 2023 for relevant articles written in English. The following search terms were used to find potential articles: “proton pump inhibitor”, “PPI”, “anti-ulcer agent”, “esomeprazole”, “lansoprazole”, “rabeprazole”, “pantoprazole”, “acute kidney injury”, “acute renal failure”, and “AKI”. All references from previous review articles were manually examined. Two independent authors scrutinized retrieved studies using pre-established selection criteria. Any disagreement during the study selection process was arbitrated by a third author and resolved by discussion.

We aimed to include both randomized controlled trials (RCTs) and observational studies, but there were no RCTs reporting PPIs and AKI risk. Therefore, in this study, we only included observational studies (such as case–control studies and cohort studies) published in English and which met the PICO (Patient Intervention Control Outcome) format. Studies were considered eligible if they met the following criteria: (a) PPI therapy was the exposure of interest; (b) AKI was the outcome; and (c) effect sizes were reported as an odds ratio (OR), hazards ratio (HR), or risk ratio (RR) with the corresponding 95% confidence interval (CI). We excluded studies published as reviews, letters, case reports, editorials, and animal studies.

### 2.2. Data Extraction

Using pre-established criteria, the same two independent reviewers extracted relevant information from the selected articles. For each study, the reviewers collected the author’s name, publication year, country, number of participants, number of PPI users, number of AKI patients, mean age, percentage of male and female patients, inclusion criteria, and effect size. Data from multiple observational studies were compiled into a single file and used to calculate the pooled effect size.

### 2.3. Risk-of-Bias Assessment

The risk of bias was assessed using the Cochrane risk-of-bias tool and the Newcastle–Ottawa Quality Assessment Scale (NOS) [27]. The risk of bias was based on the following three categories: participant selection (4 points), group comparability (2 points), and ascertainment of exposure (3 points) for case–control study or ascertainment of outcome (3 points) for cohort study. The same two authors independently assessed each study and classified it into three risk groups (low, medium, and high risk of bias) based on the scores they received out of 9 points. Any discrepancies between the two authors during the evaluation process were resolved by discussion and consultation with the third author.

### 2.4. Statistical Analysis

The meta-analysis was performed using a comprehensive meta-analysis version 3 and STATA. A random-effects model was used to calculate pooled risk ratio with the 95% CI to reduce the heterogeneity among studies [28]. A positive effect size indicates a risk effect of the PPI intervention compared with the control condition. The heterogeneity was measured using Cochran’s *Q* test. The I^2^ statistic was used to show the percentage of variability due to sampling error. The I^2^ values of 0~25%, 25~50%, 50~75%, and >75% were used to represent very low, low, moderate, and high levels of heterogeneity, respectively [29,30,31]. We drew a funnel plot and used the Egger regression test to assess publication bias, for which *p* < 0.5 indicates significant publication bias.

## 3. Results

**Figure 1:**
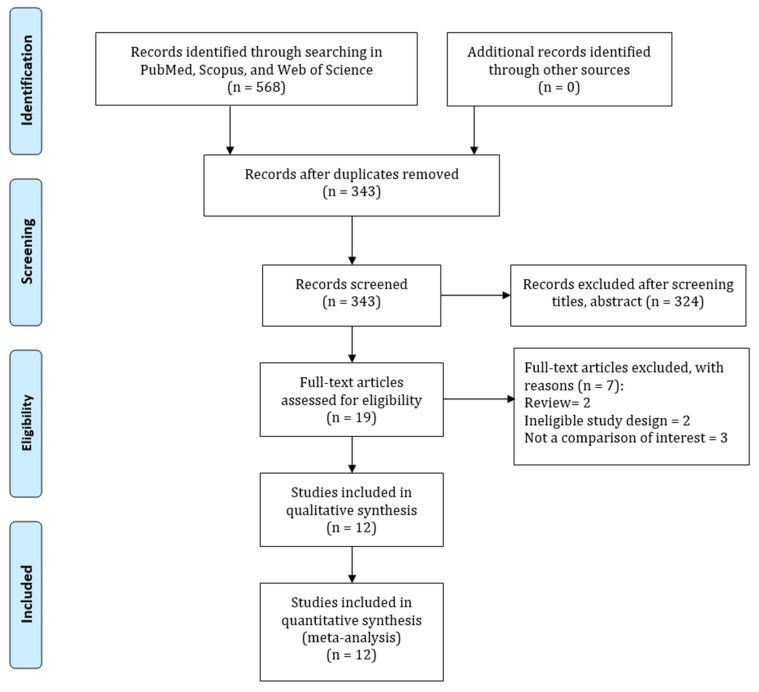
Flow chart of study selection process for the association between PPI use and acute kidney injury risk.

The electronic databases search identified 568 unique records. A total of 225 articles were removed for duplications and 19 articles were selected for full-text review after assessing titles and abstracts. Seven articles were further removed for review, as they were ineligible due to study design and inappropriate outcome of interest. Ultimately, 12 articles were included in the meta-analysis [13,21,22,23,24,32,33,34,35,36,37,38]. A visual description of the search results is available in the PRISMA flowchart in Figure 1.

### 3.1. Study Characteristics

A summary of the baseline characteristics of the included studies is presented in Table 1. Of the included twelve studies, nine were cohort [13,21,24,33,35,36,37,38] and three were case–control studies [23,32,34] involving 2,492,125 participants. Publication dates ranged from 2012 [23] and 2022 [32]. Seven studies were conducted in North America (the USA [13,22,24,34,36,38] and Canada [21]), four studies in Europe (Sweden [33], France [35], Denmark [37] and the UK [23]), and one in Asia (Japan [32]). The range of sample size was between 802 and 1,351,832. All the studies used ICD code to identify AKI patients except two studies [23,24]. The range of sample size was between 802 and 1,351,832. Half of the studies included a higher proportion of female patients, and the mean age of the patients was higher than 50 years in all studies included. Ten studies included AKI patients based on the International Classification of Disease (ICD) criteria, one study included based on the Oxford Medical Information System (OXMIS), and one study was based on the Kidney Disease Improving Global Outcomes (KDIGO).

### 3.2. Quality of Included Studies

Two cohort studies had a NOS score of 9, five studies had a score of 8 and the remaining cohort study had a score of 7. For the case–control studies, two out of three were high quality (NOS score > 7). The average NOS score was 8.07.

### 3.3. Association between PPI Use and AKI Risk

**Figure 2:**
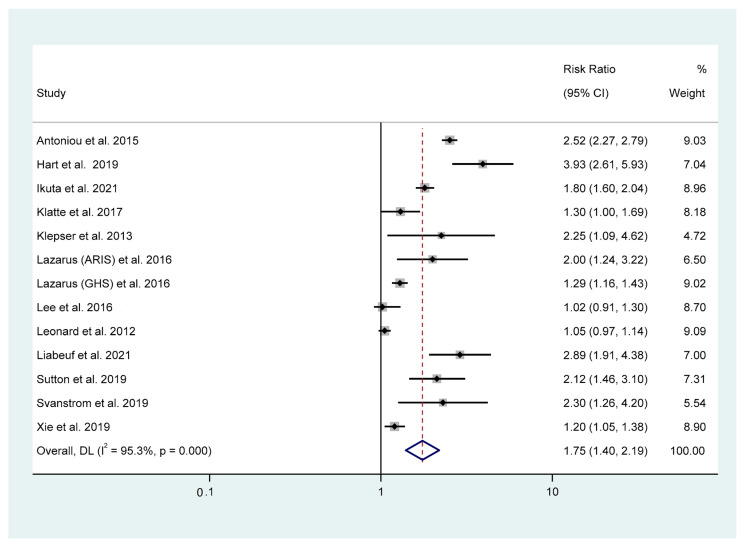
Association between PPI use and the risk of acute kidney injury [13,21,22,23,24,32,33,34,35,36,37,38].

Twelves studies were included in this meta-analysis to assess the risk of PPI use and the risk of AKI. The pooled analysis from the random effect model revealed that PPI use was associated with an increased risk of AKI (RR_adjust_ 1.75, 95%CI: 1.40–2.19, *p* < 0.001) (Figure 2). There was significant heterogeneity in the study (I^2^ = 95.3%, Q = 274.90, τ^2^ = 0.14, *p* < 0.001). 

### 3.4. Subgroup Analyses

Subgroup analysis was performed study design, study location, and methodological quality (Table 2).

Nine cohort and three case–control studies were evaluated to determine the effect of PPI use on AKI risk. The overall pooled RR estimate for cohort and case–control studies was 1.82 (95% CI: 1.38–2.41, *p* < 0.001) and 1.52 (95% CI: 0.95–2.43, *p* = 0.08), respectively. There was significant heterogeneity among the studies (I^2^ = 95.47, Q = 198.65, τ^2^ = 0.17, *p* < 0.001 and I^2^ = 96.36, Q = 55.05, τ^2^ = 0.14, *p* < 0.001)

Seven studies from North America, four studies from Europe, and one study from Asia evaluated the association between PPI and AKI risk. North American individuals had a higher risk of AKI [1.80 (95% CI: 1.31–2.48, *p* < 0.001)] compared to Asian [1.80 (95% CI: 1.59–2.03, *p* < 0.001)] and European [1.64 (95% CI: 1.06–2.54, *p* = 0.02)] individuals. There was a significant heterogeneity among studies from both North America and Europe (I^2^ = 96.26, Q = 178.36, τ^2^ = 0.18, *p* < 0.001 and I^2^ = 89.73, Q = 29.22, τ^2^ = 0.18, *p* < 0.001).

Furthermore, nine high-quality and three moderate-quality study assessed the relationship between PPI and AKI risk. The pooled risk of AKI was 1.68 (95% CI: 1.29–2.199, *p* < 0.001) in high-quality studies. However, there was a significant heterogeneity among studies (I^2^ = 96.39, Q = 249.93, τ^2^ = 0.15, *p* < 0.001). The pooled risk of AKI was 1.83 (95% CI: 1.63–2.06, *p* < 0.001) in moderate-quality studies. However, there was no significant heterogeneity among studies (I^2^ = 0, Q = 0.96, τ2 = 0, *p* < 0.001).

### 3.5. Sensitivity Analysis

As high heterogeneity was observed in the overall findings (I^2^ = 95.63%, *p* < 0.001), we therefore conducted a sensitivity analysis. To examine the overall impact of each study on AKI risk, a sensitivity analysis was performed by excluding studies one by one (Table 3). There was no significant difference in the overall effect size and the range of effect size was between 1.63 and 1.84. Moreover, the level of heterogeneity was same among the studies. 

### 3.6. Publication Bias

**Figure 3:**
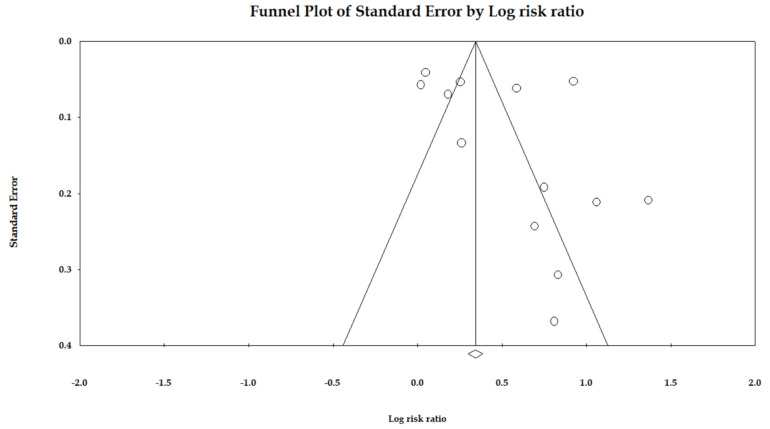
Funnel plot for the association between PPI use and the risk of acute kidney disease.

Egger’s regression was used to detect overall publication bias and also generated Begg’s funnel plots for the association between PPI use and the risk of AKI (Figure 3). It showed relatively symmetric distribution, indicating no publication bias (*p* = 0.20).

## 4. Discussion

### 4.1. Main Findings

A meta-analysis based on twelve observational studies showed that there was a significant association between PPI use and the risk of AKI. However, when stratified according to region, we found that the risk of AKI was high in North American individuals compared to Asian and European individuals. The risk of AKI was slightly lower among PPI users in high-quality studies than in moderate-quality studies. This may be due to the small number of studies focused on the association between PPI use and AKI, and the fact that some risk factors for AKI have not been fully adjusted. The findings of our study are consistent with previously published meta-analysis [39], which found that overall PPI use is associated with an increased risk of AKI. However, our meta-analysis included a higher number of studies, larger sample sizes, and comprehensive subgroup analyses based on region or methodological quality than previously published studies. The present meta-analysis is to also the first to a conduct sensitivity analysis on this topic. 

### 4.2. Biological Plausibility

Although the association between PPI use and the risk of AKI is uncertain, several possible pathophysiological mechanisms have been reported. Previous studies have shown that the development of AIN and a hypersensitivity reaction might responsible for the reducing glomerular filtration rate and adverse renal outcomes [40,41]. Moreover, PPI inhibits lysosomal activity by reducing nitric oxide synthesis and increasing hypomagnesemia, which could be another possible mechanism for increasing inflammatory and atherogenic marker secretion [42,43]. Several studies also reported that PPI use is associated with an increased risk of enteric infections, including C. difficile infection [44,45,46], and consequently also to dehydration-associated AKI [47]. Hypomagnesemia is also considered as one of the potential predictors of declining kidney function and a link to AKI [48,49]. PPI use also can increase the risk of hypomagnesemia via the disturbance of gastrointestinal handling of magnesium [50]. Clinical practice guidelines from various countries recommended the concomitant use of PPIs and NSAID to impart gastroprotection [51,52]; however, previous studies reported that the concomitant use of PPIs and NSAID might increase the risk of AKI [23,53]. Finally, PPI metabolites may deposit within the tubulointerstitial, which can lead to acute renal events and induce AIN by stimulating T-cells [54]. 

### 4.3. Clinical Implications

Kidney disease is the most common public health problem and the leading cause of death worldwide [55]. The economic burden of AKI has increased due to an increased rate of hospitalization that ranges from USD 5.4 to USD 24.0 billion [56]. The cost always varies based on the severity of the disease, and the cost is usually high if a patient requires dialysis. As the prevalence of AKI is increasing and imposing a substantial burden on our society in either both financial and psychological respects, it is therefore a top public health priority. 

While the major causes of AKI are always unclear, a number of possible risk factors for developing AKI have been identified. Previous studies reported that use of NSAIDs [57], remdesivir [58], and atypical antipsychotic [59] were associated with an increased risk of AKI. Concerns have been raised about PPI, as it is considered as a first-line, safe, and effective treatment for gastric disorders. Moreover, previous evidence also reported various adverse outcomes (e.g., dementia, hip fracture, community acquired pneumonia) associated with long-term use of PPI [15,60,61]. 

The findings of our study showed a higher risk of AKI among PPI users in three different geographical regions. Disease risk varies regionally and is always complex. Several studies have reported that physiological variation may be a factor [62,63], including genetic factors and lifestyle factors, such as eating habits, smoking, alcohol, and physical activity [64,65], as well as environmental factors, such as pollution, socioeconomic status, and stress, and access to public health services [66,67]. However, the gradual improvement of AKI symptoms, early screening, and the identification of AKI risk factors can improve the situation. However, the known and suspected risk factors cannot fully explain the risk of AKI between ethnicities.

The risk of AKI was consistent in other subgroup analyses, which makes the evidence more robust. Although PPI has been shown to have a favorable safety profile [68,69], it is important to be cautious while taking or prescribing them for patients with gastric disorders. Evidence generated from retrospective data linkage or cross-sectional studies could potentially be biased due to confounding factors, a lack of propensity score matching, and the selection of appropriate PPI users. Therefore, these findings may provide skewed risk rates. The findings of our study suggest that PPI therapy should only be used with a planned treatment strategy according to appropriate indications for a specific duration of time (based on the patient’s clinical conditions) if the overall benefits are expected to outweigh the risks.

### 4.4. Strengths and Limitations

Our study has several strengths. First, it included a larger number of individuals and studies compared to previously published meta-analysis, providing greater statistical power. Second, our study also analyzed the relationship between PPI use and the risk of AKI based on region and study quality, which was not included in previous studies. Third, to minimize the impact of potential confounding factors, our study calculated the pooled effect size using only adjusted OR/HR values, which makes our findings more robust. 

However, our study also has some limitations. First, it included only observational studies. Although most of the studies included were of high quality and adjusted for potential confounding factors, selection bias cannot be ignored. Therefore, the findings of our study cannot confirm the existence of causality. Second, we were unable to examine the risk of AKI among PPI users based on the duration of PPI use and various dosages due to data insufficiency. Third, only one study provided individual PPI use and the risk of AKI. More studies are warranted to draw a firm conclusion about different PPIs (e.g., omeprazole, rabeprazole). Fourth, our study provided a subgroup analysis based on the region but only studies from Asia were included. Therefore, more studies are needed to evaluate the regional effect. Finally, most of the included studies defined AKI based on the ICD-9 or ICD-10; however, the absolute risk of AKI in the target population can be underestimated. Two studies used laboratory data to detect AKI among PPI users; however, the effect of PPIs might be significantly affected by existed confounding factors and those studies did not categorize the stage of AKI. 

## 5. Conclusions

In this meta-analysis, we found that PPI use was associated with an increased risk of AKI. The risk of AKI was high in North American individuals compared to Asian and European individuals. Given the high prevalence of PPI use among patients with gastric disorders, safe prescription practices are necessary to ensure patients are not put at undue risk. Well-designed, with an appropriate strategy for patient selection (e.g., ICD, laboratory tests), and large prospective studies are needed in the future to gain further insight into the importance of PPI dose and duration, which can guide critical clinical decision-making. Moreover, animal studies can provide additional insight into the mechanisms by which PPI use may increase the risk of AKI.

## Figures and Tables

**Table 1:** Characteristics of studies included in the meta-analysis.

Author, Year	Location	Study Design	Sample Size	Female	Age (Mean)	AKI Assessment	Duration	NOS
Antoniou 2015 [21]	Canada	Cohort	290,592	56.7	>65	ICD	2002–2011	8
Hart 2019 [22]	USA	Cohort	93,335	61.4	51.4/50.9	ICD	1993–2008	9
Ikuta 2022 [32]	Japan	C-C	3685	36.9	52	ICD	2005–2007	7
Klatte 2017 [33]	Sweden	Cohort	114,883	60.3	62.4	ICD	2007–2010	8
Klepser 2013 [34]	USA	C-C	802	57.4	44.6	ICD	2002–2005	7
Lazarus 2016 [13]	USA	Cohort (ARIC)	11,145	57.5	62.8	ICD	1996–2011	9
		Cohort (GHS)	248,751	56.8	50.0	ICD	1997–2014	9
Leonard 2012 [23]	UK	C-C	1,351,832	50.3	68.6	OXMIS	1987–2002	8
Lee 2016 [24]	USA	Cohort	15,158	45.9	67.9	KDIGO	2001–2008	8
Liabeuf 2020 [35]	France	Cohort	3023	35	70	ICD	2013–2016	9
Sutton 2019 [36]	USA	Cohort	21,643	3	54.13	ICD	2005–2012	7
Svanstrom 2018 [37]	Denmark	Cohort	122,809	76	63	ICD	2004–2015	8
Xie 2019 [38]	USA	Cohort	21,4467	4.07	65.10	ICD	2002–2004	8

Note: C-C: case–control; AKI: acute kidney injury; ICD: international classification of disease; NOS: the Newcastle–Ottawa Scale; AIRC: Atherosclerosis Risk in Communities; GHS: Geisinger Health System; OXMIS: Oxford Medical Information System; KDIGO: Kidney Disease: Improving Global Outcomes.

**Table 2:** Subgroup analysis of the association between PPI use and the risk of acute kidney injury.

Characteristics	Number of Studies	RR (95%CI)	*p*-Value	I^2^ Value	Q Value	τ^2^	*p*-Value
All studies	12	1.75 (1.40–2.19)	<0.001	95.30	274.90	0.14	<0.001
Study design							
Cohort	9	1.82 (1.38–2.41)	<0.001	95.47	198.65	0.17	<0.001
Case–control	3	1.52 (0.95–2.43)	0.08	96.36	55.05	0.14	<0.001
Location							
North America	7	1.80 (1.31–2.48)	<0.001	96.26	187.36	0.18	<0.001
Europe	4	1.64 (1.06–2.54)	0.02	89.73	29.22	0.18	<0.001
Asia	1	1.80 (1.59–2.03)	<0.001	-	-	-	-
Methodological quality							
High	9	1.68 (1.29–2.199)	<0.001	96.39	249.93	0.15	<0.001
Moderate	3	1.83 (1.63–2.06)	<0.001	0	0.96	0	<0.001
Types of PPI							
Lansoprazole	1	2.56 (1.85–3.55)	<0.05	-	-	-	-
Omeprazole	1	2.94 (2.21–3.91)	<0.05	-	-	-	-
Pantoprazole	1	2.43 (1.97–3.00)	<0.05	-	-	-	-
Rabeprazole	1	2.45 (2.12–2.83)	<0.05	-	-	-	-

**Table 3:** Sensitivity analysis of PPI use and the risk of acute kidney injury.

Characteristics	RR (95%CI)	*p*-Value	I^2^ Value	Q Value	τ^2^	*p*-Value
Antoniou et al. [21]	1.63 (1.35–1.96)	<0.001	91.48	129.10	0.07	<0.001
Hart et al. [22]	1.63 (1.30–2.04)	<0.001	95.61	250.63	0.13	<0.001
Ikuta et al. [32]	1.74 (1.36–2.22)	<0.001	95.72	257.18	0.15	<0.001
Klatte et al. [33]	1.79 (1.41–2.26)	<0.001	95.99	274.54	0.14	<0.001
Klepser et al. [34]	1.72 (1.37–2.16)	<0.001	95.97	273.28	0.14	<0.001
Lazarus (ARIS) et al. [13]	1.72 (1.37–2.17)	<0.001	95.96	272.81	0.14	<0.001
Lazarus (GHS) et al. [13]	1.80 (1.40–2.33)	<0.001	95.95	271.69	0.17	<0.001
Lee et al. [24]	1.84 (1.45–2.33)	<0.001	95.38	238.44	0.14	<0.001
Leonard et al. [23]	1.83 (1.45–2.32)	<0.001	94.65	205.83	0.14	<0.001
Liabeuf et al. [35]	1.67 (1.33–2.10)	<0.001	95.82	263.26	0.13	<0.001
Sutton et al. [36]	1.71 (1.36–2.16)	<0.001	95.93	270.32	0.14	<0.001
Svanstrom et al. [37]	1.71 (1.36–2.15)	<0.001	95.96	272.34	0.14	<0.001
Xie et al. [38]	1.81 (1.42–2.31)	<0.001	95.91	269.09	0.15	<0.001

## Data Availability

Not applicable.

## References

[1] Nugent R.A., Fathima S.F., Feigl A.B., Chyung D. (2011). The burden of chronic kidney disease on developing nations: A 21st century challenge in global health. Nephron Clin. Pract..

[2] Namazzi R., Batte A., Opoka R.O., Bangirana P., Schwaderer A.L., Berrens Z., Datta D., Goings M., Ssenkusu J.M., Goldstein S.L. (2022). Acute kidney injury, persistent kidney disease, and post-discharge morbidity and mortality in severe malaria in children: A prospective cohort study. EClinicalMedicine.

[3] Hoste E.A., Kellum J.A., Selby N.M., Zarbock A., Palevsky P.M., Bagshaw S.M., Goldstein S.L., Cerdá J., Chawla L.S. (2018). Global epidemiology and outcomes of acute kidney injury. Nat. Rev. Nephrol..

[4] Rewa O., Bagshaw S.M. (2014). Acute kidney injury—Epidemiology, outcomes and economics. Nat. Rev. Nephrol..

[5] Silver S.A., Chertow G.M. (2017). The economic consequences of acute kidney injury. Nephron.

[6] Eriksson M., Brattström O., Mårtensson J., Larsson E., Oldner A. (2015). Acute kidney injury following severe trauma: Risk factors and long-term outcome. J. Trauma Acute Care Surg..

[7] Sun J., Sun H., Sun Z., Yang X., Zhou S., Wei J. (2021). Intra-abdominal hypertension and increased acute kidney injury risk: A systematic review and meta-analysis. J. Int. Med. Res..

[8] Patschan D., Müller G. (2016). Acute kidney injury in diabetes mellitus. Int. J. Nephrol..

[9] Abelha F.J., Botelho M., Fernandes V., Barros H. (2009). Determinants of postoperative acute kidney injury. Crit. Care.

[10] Singh P., Rifkin D.E., Blantz R.C. (2010). Chronic kidney disease: An inherent risk factor for acute kidney injury?. Clin. J. Am. Soc. Nephrol..

[11] Poly T.N., Islam M.M., Walther B.A., Lin M.-C., Li Y.-C. (2022). Proton Pump Inhibitors Use and the Risk of Pancreatic Cancer: Evidence from Eleven Epidemiological Studies, Comprising 1.5 Million Individuals. Cancers.

[12] Fass R., Sifrim D. (2009). Management of heartburn not responding to proton pump inhibitors. Gut.

[13] Lazarus B., Chen Y., Wilson F.P., Sang Y., Chang A.R., Coresh J., Grams M.E. (2016). Proton pump inhibitor use and the risk of chronic kidney disease. JAMA Intern. Med..

[14] Al-Aly Z., Maddukuri G., Xie Y. (2020). Proton pump inhibitors and the kidney: Implications of current evidence for clinical practice and when and how to deprescribe. Am. J. Kidney Dis..

[15] Islam M., Poly T.N., Walther B.A., Dubey N.K., Anggraini Ningrum D.N., Shabbir S.-A. (2018). Adverse outcomes of long-term use of proton pump inhibitors: A systematic review and meta-analysis. Eur. J. Gastroenterol. Hepatol..

[16] Sanchez-Alamo B., Cases-Corona C., Fernandez-Juarez G. (2022). Facing the challenge of drug-induced acute interstitial nephritis. Nephron.

[17] Raghavan R., Shawar S. (2017). Mechanisms of drug-induced interstitial nephritis. Adv. Chronic Kidney Dis..

[18] Brewster U., Perazella M. (2007). Proton pump inhibitors and the kidney: Critical. Clin. Nephrol..

[19] Lau J.Y., Sung J.J., Lee K.K., Yung M.-y., Wong S.K., Wu J.C., Chan F.K., Ng E.K., You J.H., Lee C. (2000). Effect of intravenous omeprazole on recurrent bleeding after endoscopic treatment of bleeding peptic ulcers. N. Engl. J. Med..

[20] Chen J., Brady P. (2019). Gastroesophageal reflux disease: Pathophysiology, diagnosis, and treatment. Gastroenterol. Nurs..

[21] Antoniou T., Macdonald E.M., Hollands S., Gomes T., Mamdani M.M., Garg A.X., Paterson J.M., Juurlink D.N. (2015). Proton pump inhibitors and the risk of acute kidney injury in older patients: A population-based cohort study. Can. Med. Assoc. Open Access J..

[22] Hart E., Dunn T.E., Feuerstein S., Jacobs D.M. (2019). Proton pump inhibitors and risk of acute and chronic kidney disease: A retrospective cohort study. Pharmacother. J. Hum. Pharmacol. Drug Ther..

[23] Leonard C.E., Freeman C.P., Newcomb C.W., Reese P.P., Herlim M., Bilker W.B., Hennessy S., Strom B.L. (2012). Proton pump inhibitors and traditional nonsteroidal anti-inflammatory drugs and the risk of acute interstitial nephritis and acute kidney injury. Pharmacoepidemiol. Drug Saf..

[24] Lee J., Mark R.G., Celi L.A., Danziger J. (2016). Proton pump inhibitors are not associated with acute kidney injury in critical illness. J. Clin. Pharmacol..

[25] Knobloch K., Yoon U., Vogt P.M. (2011). Preferred reporting items for systematic reviews and meta-analyses (PRISMA) statement and publication bias. J. Cranio-Maxillofac. Surg..

[26] Moher D., Liberati A., Tetzlaff J., Altman D.G., Group P. (2010). Preferred reporting items for systematic reviews and meta-analyses: The PRISMA statement. Int. J. Surg..

[27] Wells G., Shea B., O’connell D., Peterson J., Welch V., Losos M., Tugwell P. (2017). The Newcastle-Ottawa Quality Assessment Scale (NOS) for assessing the quality of nonrandomized studies in meta-analyses. Clin. Epidemiol. [Internet].

[28] Biggerstaff B., Tweedie R. (1997). Incorporating variability in estimates of heterogeneity in the random effects model in meta-analysis. Stat. Med..

[29] Poly T.N., Islam M.M., Walther B.A., Yang H.-C., Wu C.-C., Lin M.-C., Li Y.-C. (2020). Association between use of statin and risk of dementia: A meta-analysis of observational studies. Neuroepidemiology.

[30] Poly T.N., Islam M.M., Yang H.C., Lin M.C., Jian W.-S., Hsu M.-H., Jack Li Y.-C. (2021). Obesity and mortality among patients diagnosed with COVID-19: A systematic review and meta-analysis. Front. Med..

[31] Islam M.M., Nasrin T., Walther B.A., Wu C.-C., Yang H.-C., Li Y.-C. (2019). Prediction of sepsis patients using machine learning approach: A meta-analysis. Comput. Methods Programs Biomed..

[32] Ikuta K., Nakagawa S., Yamawaki C., Itohara K., Hira D., Imai S., Yonezawa A., Nakagawa T., Sakuragi M., Sato N. (2022). Use of proton pump inhibitors and macrolide antibiotics and risk of acute kidney injury: A self-controlled case series study. BMC Nephrol..

[33] Klatte D.C., Gasparini A., Xu H., de Deco P., Trevisan M., Johansson A.L., Wettermark B., Ärnlöv J., Janmaat C.J., Lindholm B. (2017). Association between proton pump inhibitor use and risk of progression of chronic kidney disease. Gastroenterology.

[34] Klepser D.G., Collier D.S., Cochran G.L. (2013). Proton pump inhibitors and acute kidney injury: A nested case–control study. BMC Nephrol..

[35] Liabeuf S., Lambert O., Metzger M., Hamroun A., Laville M., Laville S.M., Frimat L., Pecoits-Filho R., Fouque D., Massy Z.A. (2021). Adverse outcomes of proton pump inhibitors in patients with chronic kidney disease: The CKD-REIN cohort study. Br. J. Clin. Pharmacol..

[36] Sutton S.S., Magagnoli J., Cummings T.H., Hardin J.W. (2019). Risk of acute kidney injury in patients with HIV receiving proton pump inhibitors. J. Comp. Eff. Res..

[37] Svanström H., Lund M., Melbye M., Pasternak B. (2018). Use of proton pump inhibitors and the risk of acute kidney injury among patients with rheumatoid arthritis: Cohort study. Drug Saf..

[38] Xie Y., Bowe B., Yan Y., Xian H., Li T., Al-Aly Z. (2019). Estimates of all cause mortality and cause specific mortality associated with proton pump inhibitors among US veterans: Cohort study. BMJ.

[39] Yang Y., George K.C., Shang W.-F., Zeng R., Ge S.-W., Xu G. (2017). Proton-pump inhibitors use, and risk of acute kidney injury: A meta-analysis of observational studies. Drug Des. Dev. Ther..

[40] Simpson I.J., Marshall M.R., Pilmore H., Manley P., Williams L., Thein H., Voss D. (2006). Proton pump inhibitors and acute interstitial nephritis: Report and analysis of 15 cases. Nephrology.

[41] Raghavan R., Eknoyan G. (2014). Acute interstitial nephritis–a reappraisal and update. Clin. Nephrol..

[42] Kamal F., Khan M.A., Molnar M.Z., Howden C.W. (2018). The association between proton pump inhibitor use with acute kidney injury and chronic kidney disease. J. Clin. Gastroenterol..

[43] Corsonello A., Lattanzio F., Bustacchini S., Garasto S., Cozza A., Schepisi R., Lenci F., Luciani F., Maggio M.G., Ticinesi A. (2018). Adverse events of proton pump inhibitors: Potential mechanisms. Curr. Drug Metab..

[44] Bignardi G.E. (1998). Risk factors for Clostridium difficile infection. J. Hosp. Infect..

[45] Dial S., Alrasadi K., Manoukian C., Huang A., Menzies D. (2004). Risk of Clostridium difficile diarrhea among hospital inpatients prescribed proton pump inhibitors: Cohort and case–control studies. CMAJ.

[46] Leonard J., Marshall J.K., Moayyedi P. (2007). Systematic review of the risk of enteric infection in patients taking acid suppression. Off. J. Am. Coll. Gastroenterol. ACG.

[47] Arrich J., Sodeck G.H., Sengölge G., Konnaris C., Müllner M., Laggner A.N., Domanovits H. (2005). Clostridium difficile causing acute renal failure: Case presentation and review. World J. Gastroenterol. WJG.

[48] Santos B., Seguro A., Andrade L. (2010). Hypomagnesemia is a risk factor for nonrecovery of renal function and mortality in AIDS patients with acute kidney injury. Braz. J. Med. Biol. Res..

[49] Alves S.C., Tomasi C.D., Constantino L., Giombelli V., Candal R., Bristot M.d.L., Topanotti M.F., Burdmann E.A., Dal-Pizzol F., Fraga C.M. (2013). Hypomagnesemia as a risk factor for the non-recovery of the renal function in critically ill patients with acute kidney injury. Nephrol. Dial. Transplant..

[50] Van Laecke S., Nagler E.V., Verbeke F., Van Biesen W., Vanholder R. (2013). Hypomagnesemia and the risk of death and GFR decline in chronic kidney disease. Am. J. Med..

[51] Tannenbaum H., Peloso P., Russell A., Marlow B. (2000). An evidence-based approach to prescribing NSAIDs in the treatment of osteoarthritis and rheumatoid arthritis: The Second Canadian Consensus Conference. Can. J. Clin. Pharmacol. J. Can. Pharmacol. Clin..

[52] Members W.C., Bhatt D.L., Scheiman J., Abraham N.S., Antman E.M., Chan F.K., Furberg C.D., Johnson D.A., Mahaffey K.W., Quigley E.M. (2008). ACCF/ACG/AHA 2008 expert consensus document on reducing the gastrointestinal risks of antiplatelet therapy and NSAID use: A report of the American College of Cardiology Foundation Task Force on Clinical Expert Consensus Documents. Circulation.

[53] Ikuta K., Nakagawa S., Momo K., Yonezawa A., Itohara K., Sato Y., Imai S., Nakagawa T., Matsubara K. (2021). Association of proton pump inhibitors and concomitant drugs with risk of acute kidney injury: A nested case–control study. BMJ Open.

[54] Moledina D.G., Perazella M.A. (2016). PPIs and kidney disease: From AIN to CKD. J. Nephrol..

[55] Perico N., Remuzzi G. (2012). Chronic kidney disease: A research and public health priority. Nephrol. Dial. Transplant..

[56] Silver S.A., Long J., Zheng Y., Chertow G.M. (2017). Cost of acute kidney injury in hospitalized patients. J. Hosp. Med..

[57] Nash D.M., Markle-Reid M., Brimble K.S., McArthur E., Roshanov P.S., Fink J.C., Weir M.A., Garg A.X. (2019). Nonsteroidal anti-inflammatory drug use and risk of acute kidney injury and hyperkalemia in older adults: A population-based study. Nephrol. Dial. Transplant..

[58] Wong C.K., Au I.C., Cheng W.Y., Man K.K., Lau K.T., Mak L.Y., Lui S.L., Chung M.S., Xiong X., Lau E.H. (2022). Remdesivir use and risks of acute kidney injury and acute liver injury among patients hospitalised with COVID-19: A self-controlled case series study. Aliment. Pharmacol. Ther..

[59] Hwang Y.J., Dixon S.N., Reiss J.P., Wald R., Parikh C.R., Gandhi S., Shariff S.Z., Pannu N., Nash D.M., Rehman F. (2014). Atypical antipsychotic drugs and the risk for acute kidney injury and other adverse outcomes in older adults: A population-based cohort study. Ann. Intern. Med..

[60] Haenisch B., von Holt K., Wiese B., Prokein J., Lange C., Ernst A., Brettschneider C., König H.-H., Werle J., Weyerer S. (2015). Risk of dementia in elderly patients with the use of proton pump inhibitors. Eur. Arch. Psychiatry Clin. Neurosci..

[61] Poly T., Islam M., Yang H.-C., Wu C., Li Y.-C. (2019). Proton pump inhibitors and risk of hip fracture: A meta-analysis of observational studies. Osteoporos. Int..

[62] Kopitkó C., Medve L., Gondos T., Soliman K.M.M., Fülöp T. (2022). Mediators of Regional Kidney Perfusion during Surgical Pneumo-Peritoneum Creation and the Risk of Acute Kidney Injury—A Review of Basic Physiology. J. Clin. Med..

[63] Murugan R., Kellum J.A. (2011). Acute kidney injury: What’s the prognosis?. Nat. Rev. Nephrol..

[64] Kashani K., Macedo E., Burdmann E.A., Hooi L.S., Khullar D., Bagga A., Chakravarthi R., Mehta R., Group C., Initiative A.D.Q. (2017). Acute kidney injury risk assessment: Differences and similarities between resource-limited and resource-rich countries. Kidney Int. Rep..

[65] Jha V., Garcia-Garcia G., Iseki K., Li Z., Naicker S., Plattner B., Saran R., Wang A.Y.-M., Yang C.-W. (2013). Chronic kidney disease: Global dimension and perspectives. Lancet.

[66] Mohammad K.N., Chan E.Y.Y., Lau S.Y.-F., Lam H.C.Y., Goggins W.B., Chong K.C. (2021). Relationship between acute kidney injury, seasonal influenza, and environmental factors: A 14-year retrospective analysis. Environ. Int..

[67] McTavish R.K., Richard L., McArthur E., Shariff S.Z., Acedillo R., Parikh C.R., Wald R., Wilk P., Garg A.X. (2018). Association between high environmental heat and risk of acute kidney injury among older adults in a northern climate: A matched case-control study. Am. J. Kidney Dis..

[68] Nguyen P.A., Islam M., Galvin C.J., Chang C.-C., An S.Y., Yang H.-C., Huang C.-W., Li Y.-C., Iqbal U. (2020). Meta-analysis of proton pump inhibitors induced risk of community-acquired pneumonia. Int. J. Qual. Health Care.

[69] Johnstone J., Nerenberg K., Loeb M. (2010). Meta-analysis: Proton pump inhibitor use and the risk of community-acquired pneumonia. Aliment. Pharmacol. Ther..

